# Development of *Agave* as a dedicated biomass source: production of biofuels from whole plants

**DOI:** 10.1186/s13068-015-0261-8

**Published:** 2015-05-30

**Authors:** Jonathan R Mielenz, Miguel Rodriguez, Olivia A Thompson, Xiaohan Yang, Hengfu Yin

**Affiliations:** Biosciences Division, Oak Ridge National Laboratory, One Bethel Valley Rd., PO Box 2008, Oak Ridge, TN 37831 USA; White Cliff Biosystems Co., 528 Pointe Vista Dr., Rockwood, TN 37854 USA

**Keywords:** *n*-Butanol, Butyric acid, Ethanol, *Clostridium beijerinckii*, Dedicated feedstock, Fermentation, Inulinase, Poly-fructose, Semi-arid

## Abstract

**Background:**

*Agave* species can grow well in semi-arid marginal agricultural lands around the world. Selected *Agave* species are used largely for alcoholic beverage production in Mexico. There are expanding research efforts to use the plentiful residues (bagasse) for ethanol production as the beverage manufacturing process only uses the juice from the central core of mature plants. Here, we investigate the potential of over a dozen *Agave* species, including three from cold semi-arid regions of the United States, to produce biofuels using the whole plant.

**Results:**

Ethanol was readily produced by *Saccharomyces cerevisiae* from hydrolysate of ten whole *Agaves* with the use of a proper blend of biomass degrading enzymes including inulinase that overcomes inhibition of most of the species tested. As an example, US grown *Agave neomexicana* produced 119 ± 11 mg ethanol/g biomass. Unlike yeast fermentations, *Clostridium beijerinckii* produced *n*-butanol plus acetone from all species tested. Butyric acid, a precursor of *n*-butanol, was also present due to incomplete conversion during the screening process. Since *Agave* contains high levels of free and polyfructose which are readily destroyed by acidic pretreatment, a two-step procedure was developed to depolymerize polyfructose while maintaining its fermentability. The hydrolysate from before and after dilute acid processing was used in *C. beijerinckii* fermentations with selected *Agave* species with *A. neomexicana* producing 144 ± 4 mg fermentation products/g biomass.

**Conclusions:**

Results showed *Agave*’s potential to be a source of fermentable sugars beyond the existing beverage species to now include many species previously unfermentable by yeast, including cold-tolerant lines. This development should stimulate development of *Agave* as a dedicated feedstock for biofuels in semi-arid regions throughout the globe.

**Electronic supplementary material:**

The online version of this article (doi:10.1186/s13068-015-0261-8) contains supplementary material, which is available to authorized users.

## Background

*Agave* is a succulent plant primarily native to Mexico and Central America that was spread across the globe by the Spanish and Portuguese, among others, to the Mediterranean coast, and eventually East Africa, the Philippines, Indonesia, Australia, and elsewhere. While there are hundreds of species of *Agave* [1, 2], *Agave americana*, *Agave angustifolia*, *Agave fourcroydes*, and *Agave sisalana* were among the species commonly distributed across the warmer areas of the world, especially during the nineteenth century. Historical interest in *Agave* was due to its multiple uses for food, animal feed, fiber, ornamental beauty, and in Mexico, for alcoholic beverages [1, 3].

*Agave* is a very efficient plant regarding water use because of its crassulacean acid metabolism (CAM). This adaptation minimizes *Agave*s’ water loss by limiting transpiration during the heat of the day by closing the plant stomata. As a result, carbon dioxide fixation occurs predominately during the cooler night when the stomata are open [4]. So, *Agave* species are well adapted to arid and semi-arid environments around the world which includes its homeland Mexico and the southwestern United States. However, this metabolic strategy does not limit *Agave* plants productivity under semi-arid conditions since young *Agave tequilana* have been estimated to produce 21.1–24.9 Mg/ha/year in Jalisco, Mexico [5], which compares well with the 10-year average of 22.8 Mg/ha/year for switchgrass *Panicum virgatum* grown in Alabama in the US [6] under non-semi-arid conditions. Therefore, *Agave* can be sufficiently productive even with only modest rainfall common to portions of Mexico and the western United States.

Production of fermented beverages from *Agave* as well as its use for food results from the plant’s high soluble non-structural carbohydrate content [3]. During growth-employing CAM metabolism, glucose is produced and converted to other sugars by the pentose phosphate pathway and sugar metabolism which results in high levels of fructose and sucrose. Also, as an energy reserve, *Agave* produces a variety of inulin-like storage polymers of fructose [7–9] which are distributed throughout the whole *Agave* plant [10]. Furthermore, analysis of published research [11] has shown that *A. americana* and *Agave salmiana* have as much as ~54 to 57 % of their total carbohydrates in the form as soluble sugar while *A. tequilana* contained only ~34 % of their carbohydrates as soluble carbohydrates. The challenge is to utilize all the fermentable carbohydrates in *Agave* for biofuels production.

The juxtaposition of a need for bioenergy feedstock from marginal lands such as much of the western United States with the clear potential for *Agave* as a source of readily fermentable simple sugars has spurred a strong interest in use of *Agave* for bioenergy [4, 12–19]. Progress has been made using process residues from the manufacture of *Agave* beverages and other products from biofuel production [3, 17, 20–22]. However, to attain large-scale production of biofuels such as ethanol from *Agave,* additional non-beverage species able to grow in a broader agronomic range need to be used. To this end, here we show research results of screening whole container-grown *Agave* plants from multiple species for their capability to produce either ethanol (thirteen species) or *n*-butanol plus acetone (nine species), including *Agave*, able to be cultivated in the United States.

## Results and discussion

### SSF production of ethanol

**Fig. 1:**
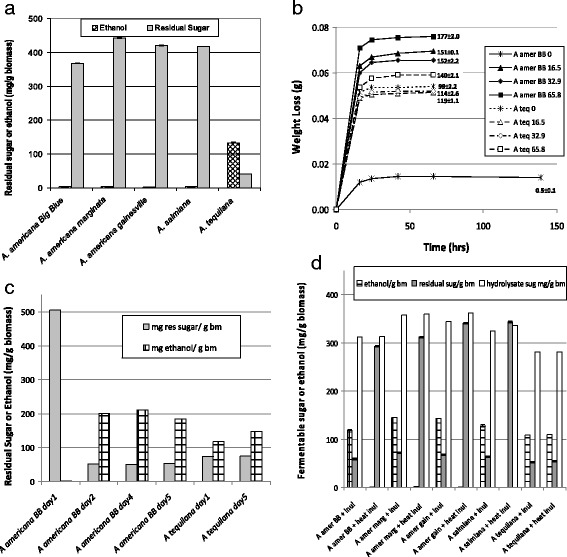
Ethanol fermentation of unpretreated Agave biomass with and without active inulinase present during the hydrolysis by a cellulase cocktail. **a** SSF fermentation of five unpretreated *Agave* biomass without inulinase: ethanol and residual sugar after 35 °C 304-hour fermentation as mg/g dry biomass. *n* = 3 with standard deviation. **b** Weight loss during SHF ethanol 35 °C production of unpretreated *A. americana* BB and *A. tequilana* biomass with different levels of inulinase. *Numbers* are ethanol mg/g dry biomass with standard deviation. *Legend numbers* are the level of inulinase added (INU/g biomass) with inulinase activity of 329 INU/mL (see “Methods” section). *n* = 2. **c** Impact of time on enzymatic hydrolysis with inulinase prior to ethanol fermentation of two unpretreated *Agave* biomass. Ethanol level and residual sugar after 35 °C 48-hour fermentation: mg/g dry biomass. Identical hydrolysis tests were initiated and one removed on days indicated and frozen waiting analysis. **d** 35 °C 42-hour SHF conversion of five unpretreated *Agave* with addition of native or heat killed inulinase including in the hydrolysis step. Starting sugar levels, ethanol levels, and remaining residual sugars is mg/g dry biomass with standard deviation. *n* = 2; *inul* inulinase enzyme; heat heated inulinase enzyme, *A amer BB A. americana* Big Blue, *A amer marg A. americana* marginata, *A amer gain A. americana* gainesville

As mentioned, the goal of this research is to evaluate the potential of multiple *Agave* species as a biofuels feedstock by using the whole plant as opposed to use of bagasse after extraction of readily solubilized sugars for beverage production [3, 20, 22]. Here, the results of fermentation of the whole *Agave* biomass are described using both yeast for ethanol production and an improved strain of *Clostridium beijerinckii* (BA101) for production of *n*-butanol plus acetone. Due to the high levels of soluble carbohydrates in *Agave* including polyfructose molecules found in all portions of *Agave* [10], the whole *Agave* was milled and used directly, unpretreated, for enzymatic hydrolysis and fermentation using well-established methods [23]. Initial fermentations used simultaneous saccharification and fermentation (SSF) with addition of enzymes and yeast together during the fermentation at temperatures favorable for the yeast. These SSF tests used biomass from five *Agave* species: *A. americana* Big Blue (BB), *A. americana* var. *marginata*, *A. americana* var. *gainesville*, *A. salmiana*, and *A. tequilana.* The fermentations were largely unsuccessful as shown in Fig. 1a except for *A tequilana*. The results show that simple sugars were released by the action of cellulases and other enzymes allowing *A. tequilana* to produce 132.9 mg ethanol/g biomass. Comparatively, a negligible amount of ethanol (2.2–2.7 mg ethanol/g biomass) was produced from the other four species. The carbohydrase enzymes were active as there were high levels of residual unfermented sugars (368–443 mg/g biomass for glucose, galactose, mannose, and fructose) for the unsuccessful fermentations suggesting the yeast fermentation was inhibited by unknown substances for the non-*A tequilana* species. A number of chemical extractions were performed and evaluated including anionic, cationic, and hydrophobic extractions in addition to activated carbon sorption. These tests yielded improvement in fermentation in combination with reduced sugar availability (data not shown). Control experiments showed no ethanol production from components in the enzyme preparation when biomass was excluded during the hydrolysis (data not shown).

### Role of inulinase in SHF ethanol production

Fortunately, as part of the search for improved fermentability of the non-*A tequilana* species, an additional enzyme, *Aspergillus niger* inulinase from Novozymes (Sigma-Aldrich Chemical Co., St. Louis, MO, USA) was tested since inulinase is known to readily hydrolyze polyfructose polymers found in chicory and many other plants including *Agave* which contains inulin-like fructose polymers [7–10]. Our research with other biomass feedstocks showed separate hydrolysis and fermentation (SHF) was preferable for *n*-butanol fermentations (unpublished observations) which were planned with *Agave*, so SHF conversions were used for further work unless stated otherwise. SHF involves hydrolysis of the biomass by biomass carbohydrate degrading enzymes at temperatures favorable for the enzymes, typically ≥50 °C, followed by fermentation of the hydrolysate with the selected microorganism at their preferred temperature. *Agave* hydrolysates that included inulinase demonstrated dramatic improvement in yeast fermentation compared to those without inulinase. The impact of different levels of added inulinase on hydrolysis of *A. americana* BB is shown in Fig. 1b with levels ranging from zero inulinase to 65.8 inulinase units (INU) per gram *Agave* biomass. The fermentation progress was monitored by venting the bottle fermentations as described previously [23]. While these enzyme levels were high, it was clear that addition of as low as 16.5 INU/g biomass yielded a hydrolysate that produce 151 ± 0.1 mg ethanol/g biomass while failure to add inulinase resulted in very low ethanol yields (0.5 ± 0.1 mg/g biomass) even after additional 3 days of fermentation. *A. tequilana* hydrolysate was fermented successfully with all conditions producing between 120 and 140 mg ethanol/g biomass with inulinase levels from zero to 65.8 INU/g biomass, again demonstrating no inhibition of fermentation with this species of *Agave*.

With the addition of inulinase being critical to yield a fermentable hydrolysate, the time required for hydrolysis was tested by limiting the time of hydrolysis from 1 to 5 days, followed by fermentation. As shown in Fig. 1c, exposure of the *A. americana* biomass to the enzyme cocktail with inulinase at 32.9 INU/g biomass required approximately 2 days to produce a fermentable hydrolysate. Hydrolysis with inulinase for 1 day yielded no ethanol but approximately 505 mg fermentable sugar, and for day 2 through day 5, ethanol levels varied from 185 to 211 mg/g biomass. Clearly, the lack of ethanol production was not due to the lack of fermentable sugars at day 1. *A. tequilana* did not show this delay in fermentation yielding ethanol at day 1. Further hydrolysis was conducted for a minimum of 4 days to obtain the maximum benefit of the inulinase enzyme addition.

It was important to determine if the impact of the inulinase was likely due to enzymatic activity, and *Aspergillus* inulinase is known to be inactivated at temperatures 75 °C or above [24, 25]. Therefore, the inulinase enzyme preparation from Novozymes was used for hydrolysis either untreated or after heating to 85 °C for 30 min. As shown in Fig. 1d, native and heated inulinase was included in the hydrolysis of biomass from five *Agave* species. Inclusion of heated inulinase during the hydrolysis for four non-*tequilana Agave* species again yielded no fermentation but significant biomass hydrolysis evidenced by the residual sugars present. When native inulinase was added during the hydrolysis, fermentation of the sugars to ethanol proceeded similarly to *A. tequilana,* which has been shown not to require added inulinase.

### Ethanol production from multiple *Agave* species

**Fig. 2:**
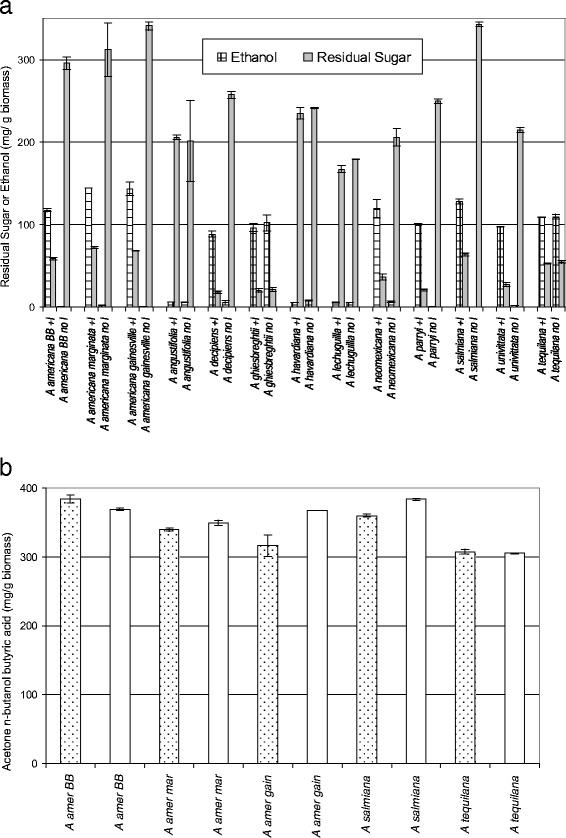
Fermentation results after SHF conversion of multiple *Agave* species for **a** ethanol production or **b** acetone, *n*-butanol, and butyric acid (ABB) production. **a** Thirteen unpretreated *Agave* were hydrolyzed with a enzyme mixture with or without inulinase present. Residual sugar and fermentation products are mg/g dry biomass with standard deviation. *+I* added inulinase, *no I* no added inulinase. Thirty-five-degree Celsius fermentation time varied from 42 to 164 hours depending upon the time the weight loss ceased. *n* = 2 **b** ABB production from five unpretreated *Agave* with native or heat killed inulinase during SHF conversion. *Hatched bar*: native/active inulinase added; *Clear bar*: heat-inactivated inulinase added. The 35 °C 162-hour fermentation, *n* = 2. ABB is mg/g dry biomass with standard deviation. *amer americana;* BB big blue; *marg marginata*; gain gainesville

As part of the screening of multiple *Agave* species, 13 species shown in Table 1 were tested by SHF processing with and without added inulinase. Results of SHF processing are shown in Fig. 2a, and these results can be summarized into three classes. The first class is *Agave* species that require inulinase for effective fermentation by yeast which include all three *A. americana* varieties along with *Agave decipiens, Agave neomexicana, Agave parryi*, *A. salmiana*, and *Agave univitatta*. The second class has two species which did not require inulinase for ethanol fermentation: *Agave ghiesbreghtii* and *A. tequilana*. Surprisingly, the third class has three species which did not demonstrate ethanol fermentation with addition of the standard level of inulinase (see “Methods” section) in the hydrolysis: *A. angustifolia, Agave havardiana*, and *Agave lechuguilla* presumably due to toxins in the hydrolysate that were not sufficiently detoxified by the added inulinase. In some cases, there was a moderate level of unfermented sugars in fermentations indicating this screening approach will benefit from future SHF optimization. However, the use of inulinase in yeast fermentations has been shown to be widely effective for most of the *Agave* species tested, including one species, *A. neomexicana*, which yielded 119 ± 11 mg ethanol/g biomass (Fig. 2a) and has significant cold tolerance to −29 °C, as shown in Table 1.

**Table 1:** Harvest wet weight, US/regional source and cold hardiness of 13 *Agave* species

*Agave* species	Whole plant harvest wet wt (g)	US States, territory^a^	Cold hardiness (°C)^b^
*Agave americana* var*.* Big Blue	1829.4	CA; AZ; TX; LA; FL; HI; VI	−9
*Agave americana* var*. marginata*	2559.8		−7^b^
*Agave americana* var*. gainesville*	2503.7		−9^b^
*Agave angustifolia*	70.1	Imported	−4
*Agave decipiens*	89.6	FL	−1^b^
*Agave ghiesbreghtii*	169.4	Imported	−4
*Agave havardiana*	549.5	NM; TX	−23
*Agave lechuguilla*	1223.8	TX	−18
*Agave neomexicana*	155.9	AZ; TX	−29
*Agave parryi* var*. truncate*	72.2	AZ; NM; TX	−9
*Agave salmiana* var*. ferox*	2580.8	Imported	−4
*Agave tequilana*	1708.6	Imported	−4
*Agave univitatta* var*. compacta*	163.6	AZ; TX	−7

^a^Locations from a USDA database (http://plants.usda.gov/java/nameSearch)

^b^Cold hardiness: J Notestein, personal communication; others from [2]

The nature of the impact of the inulinase enzyme preparation on hydrolysis and fermentation is not known. One option could be that the *Saccharomyces cerevisiae* D5A was particularly sensitive to the composition of the enzyme hydrolysate. To determine if another yeast was impacted by *Agave* hydrolysate, *Saccharomyces bayanus* EC118, a commercial champagne yeast capable of high ethanol production and fermentation up to about 32 °C, was tested in SHF conversion. The fermentation of *A. americana* var. *marginata* hydrolysate was tested with this yeast either with or without inulinase addition during the hydrolysis process. Ethanol production occurred only with addition of inulinase, yielding 141 ± 0.8 mg ethanol/g biomass, while in the absence of inulinase, the hydrolysate after fermentation yielded 8.0 ± 0.7 mg ethanol/g biomass and high levels of residual unfermented sugar. These results showed the sensitivity to hydrolysate that did not include inulinase is not specific for *S. cerevisiae* D5A.

### *C. beijerinckii* acetone *n*-butanol fermentation

*n*-Butanol is an important biofuel whose fermentation production has been well developed using glucose and sucrose fermentation with various *Clostridium* species [26]. Prof. Hans Blaschek developed hyper-*n*-butanol-producing mutant *C. beijerinckii* BA101 [27, 28] that produces acetone and *n*-butanol but essentially no ethanol during fermentation. This strain was used to ferment the hydrolysate that was used for yeast with native or heated inulinase included in the hydrolysis step (Fig. 1c). As shown in Fig. 2b, *C. beijerinckii* was able to ferment all the samples for the three *A. americana* varieties, *A. salmiana*, and *A. tequilana* regardless of the addition of active inulinase yielding similar levels of acetone and *n*-butanol per gram of *Agave* biomass used. An example of the time course fermentation for three species is shown in Additional file 1: Figure S1. The levels of acetone plus *n*-butanol ranged from 210 to 289 mg/g biomass with 62–118 mg butyric acid/g biomass, depending upon species. Butyric acid results from incomplete conversion to *n*-butanol. Figure 2b and Additional file 2: Table S1 show that there is no consistent pattern regarding the benefit of the inulinase. Therefore, the inhibitory substance that affected the yeast fermentation had no impact upon *C. beijerinckii* BA101 and the fermentation. Similar results were found for *A. univitatta*, *A. parryi, A. neomexicana, A. havardiana,* and *A. lechuguilla* (data not shown). The hydrolysates from the last two species were not successfully fermented by yeast, yet the sugars in this hydrolysate were successfully fermented by *C. beijerinckii* BA101 to acetone, *n*-butanol, and butyric acid.

### Selective hydrolysis and fermentation of *Agave* fructans

**Fig. 3:**
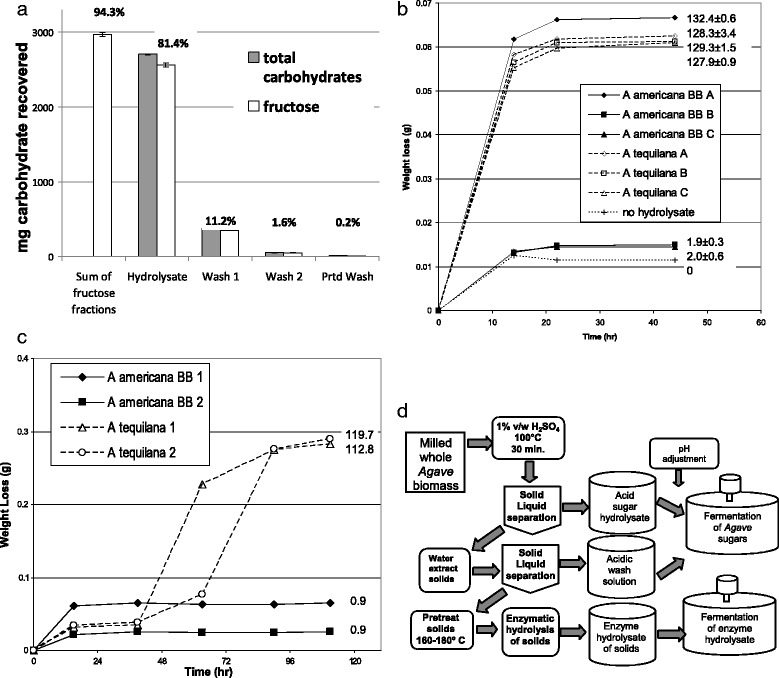
Development of a two stage conversion procedure for improved conversion of *Agave* carbohydrates. **a** Shown are fructose and total carbohydrate levels after a mild acidic hydrolysis process of a blend of 3 g each cellulose and inulin in triplicates. The total milligrams of fructose or total carbohydrates liberated are shown with standard deviation. *Inserted number*: percentage of fructose recovered in relevant fraction. Fructose percent data was corrected for hydrolysis weight gain. **b** Impact of acid hydrolysis of two *Agave* biomass sources with and without inulinase on SHF conversion to ethanol. All samples received acid hydrolysis before enzymatic hydrolysis with or without inulinase. *A* Room temperature acid hydrolysis with added inulinase. *B* High temperature acid hydrolysis without added inulinase. *C* Room temperature acid hydrolysis without added inulinase. Weight loss of individual duplicate 35 °C fermentations is shown. **c** Impact of high temperature acid hydrolysis of two *Agave* biomass sources before SSF enzymatic hydrolysis and fermentation with and without inulinase. Weight loss of individual duplicate 35 °C fermentations is shown. *Solid line A. americana* BB biomass, *dotted line A. tequilana* biomass. *Numbers* in (**b**) and (**c**) are ethanol mg/g dry biomass with standard deviation. **d** Two-step *Agave* sugar extraction and fermentation procedure using mild acid hydrolysis, solid washing, and acidic pretreatment of solids followed by enzymatic hydrolysis yielding two fermentation sugar streams

Commercial inulinase preparations act by cleaving d-fructosidic linkages. They include endo-inulinases (EC 3.2.1.7) or exo-inulinases (EC 3.2.1.80) [29]. *Agave* contains multiple forms of polyfructose molecules [7, 8] that should be sensitive to inulinase to varying degrees. Hydrolysis of polyfructose will liberate fructose which is readily fermentable by yeast. However, it is not clear why inulinase activity would be required for fermentation since in the absence of inulinase, the fermentation broth contains ample glucose, galactose, and mannose, all fermentable by yeast. If the inhibition was due to polyfructose molecules, *A. tequilana* could be expected to be similarly impacted without inulinase hydrolysis of the polyfructans. The results in Fig. 1d show the mechanism of removal of inhibition is heat sensitive, therefore possibly enzyme based. Therefore, an acid hydrolysis method was developed to determine if acidic hydrolysis of the polyfructans to fructose alone removed the inhibition. Using results from quantification of fructose in herbaceous biomass [30], an initial step was exposure of the *Agave* biomass to 1 % sulfuric acid at 100 °C for 30 min. Interestingly, a recent publication [31] arrived at the similar conditions independently for *Agave* leaf juice hydrolysis. The efficacy of this approach was tested by hydrolyzing a mixture of 3 g inulin (#I2255; Sigma-Aldrich, St. Louis, MO, USA) plus 3 g cellulose (FMC Avicel pH-105; FMC Corporation, Philadelphia, PA, USA) with these conditions, followed by washing of the solids. The liquid fractions were examined for fructose levels by HPLC and the results are shown in Fig. 3a. The inulin was nearly completely recovered as fructose at a ~94 % level with about ~81 % of the 3 g of inulin in the 1 % acid solution. The first wash of the solids contained an additional ~11 % of the fructose, with about 2 % in the second wash plus pretreatment wash. The hydrolysis method resulted in liberation of only 5 % of the glucose from the 3 g cellulose solids due to the mild temperature treatment that fully hydrolyzed the solid inulin. These results show this test method is effective in selectively hydrolyzing polyfructose molecules while leaving structural cellulose etc. nearly intact.

To test this acid hydrolysis method on *Agave*, biomass from *A. americana* BB and *A. tequilana* was hydrolyzed with 1 % sulfuric acid, without and with heating as described above, to depolymerize polyfructose molecules. Following neutralization of the biomass, two fermentations schemes, SHF and SSF, were used in tests comprising four conditions. The first three used SHF: (A) hydrolysis including inulinase of the unheated (room temperature)-acid-treated biomass followed by fermentation, (B) hydrolysis without inulinase of hot-acid-treated biomass followed by fermentation, (C) hydrolysis without inulinase of the unheated-acid-treated biomass followed by fermentation. The fourth condition was SSF processing of hot-acid-treated biomass without inulinase. Therefore, all the samples were subjected to acid hydrolysis, and two at elevated temperatures, one for SSF and one for SHF processing. Figure 3b, c shows the results of SHF and SSF conversion by yeast, respectively. For *A. americana* BB samples, only fermentations that contained the inulinase during hydrolysis produced ethanol yielding 132.4 ± 0.6 mg ethanol/g biomass by SHF conversion (Fig. 3b). Neither the high temperature nor the room temperature acid treated samples yielded significant ethanol (1.9 ± 0.3 and 2.0 ± 0.6 mg/g biomass) after fermentation, but contained ample free fermentable sugars (glucose, fructose, galactose, and mannose). However, *A. tequilana* biomass sugars were readily fermented to ethanol regardless of the conditions, yielding an average of 128.5 ± 0.7 mg ethanol/g biomass for all three conditions, showing the methods used to process the samples produced fermentable sugars.

Figure 3c shows the progress of individual SSF conversions which did not include inulinase. *A. americana* BB SSF conversion produced only 0.9 ± 0.02 mg ethanol/g biomass. SSF conversion of similar treated *A. salmiana* biomass showed the same result (data not shown). However *S. cerevisiae* readily fermented the *A. tequilana* sugars in SSF mode producing 116.3 ± 4.9 mg ethanol/g biomass. These results show the hydrolysis of the polyfructose alone is not the cause of improve fermentability of *Agave* sugars but rather some other inhibitor or toxin is responsible for the poor fermentability. Since SHF and SSF processes yielded similar results for *A. tequilana*, the fermentation method (SSF vs SHF) is not the cause of poor fermentation without inulinase for most *Agave* species. These results suggest there is an additional heat-labile agent, likely enzymatic, in the Novozymes inulinase that is responsible for improved fermentability of the *Agave* sugars, but only after about 2 days incubation under the test conditions used.

### Two-step fermentation procedure

With successful fermentation of the majority of the *Agave* by both yeast with inulinase addition and by *C. beijerinckii* BA101, an improved approach was required as the structural solids containing cellulose and hemicellulose are poorly accessed by direct enzymatic hydrolysis without pretreatment, which is known to improve enzyme accessibility [32]. Unfortunately, direct pretreatment of the *Agave* biomass by high-temperature approaches, which were likely to be acidic due to acetate and other organic acids in *Agave*, would destroy the fructose [30]. Therefore, a two-step procedure was developed with a first step that allowed hydrolysis of *Agave* polyfructose without destruction of the fructose. Using a previously test method (Fig. 3a), the biomass was hydrolyzed with relatively mild conditions of 100 °C 1 % *w*/*w* H_2_SO_4_ for 30 min to selectively hydrolyze the polyfructose molecules, leaving structural cellulose largely unhydrolyzed as previously shown with inulin and cellulose tests. In the process shown in Fig. 3d, the solids in the acid hydrolysate are washed to remove residual soluble sugars, and the solids are subjected to a pretreatment step. While there are numerous pretreatment options [32], dilute acid hydrolysis at 170 °C for 10 min was chosen for these *Agave* tests using only the residual acid present in the solids from step 1. The solids were used for enzymatic hydrolysis to provide fermentable sugars. The two-step scheme (Fig. 3d) shows two fermentations, but they can be combined if desired, minimizing fermentation vessels.

**Fig. 4:**
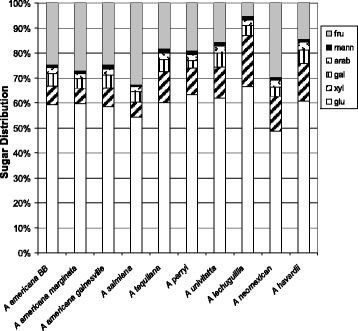
Percentage distribution of simple sugars in ten *Agave* after processing with the two-step procedure (Fig. 3d). *fru* fructose, *mann* mannose, *arab* arabinose, *gal* galactose, *xyl* xylose, *glu* glucose

The sugar distribution of ten *Agave* species after hydrolysis using the two-step process (Fig. 3d) is shown in Table 2. The sugars evaluated were glucose, xylose, arabinose, fructose, galactose, and mannose. There were considerable differences in the fraction content by species with three *A. americana* varieties, *A. salmiana*, *A. parryi*, and *A. neomexicana* having about 40 % or more of their sugars available after mild hydrolysis (step 1). *A. tequilana*, whose hydrolysates were readily fermentable by both yeast and *C. beijerinckii*, has about two-thirds of its carbohydrates as solids like *A. univitatta* and *A. havardiana*. Indeed, during handling, *A. tequilana* solids appeared more fibrous than other samples possible due to higher levels of structural carbohydrates. The individual sugar content is shown in detail in Fig. 4 and Additional file 3: Table S2 with all the species having glucose content at or over ~50 % of the total carbohydrates analyzed, generated primarily from enzyme hydrolysis of the solids (Table 2). Fructose is an important carbohydrate in most *Agave*, but it is readily destroyed if the biomass is exposed to extreme heating and acidic conditions such as those used in dilute-acid pretreatment. The content of fructose in the *Agave* species, which is largely only present in the mild acid hydrolysate, ranged from 25 to 33 % of the total evaluated sugars for the *A. americana* varieties, *A. neomexicana*, and *A. salmiana* to the very low level found in *A. lechuguilla* (Fig. 4 and Additional file 3: Table S2).

**Table 2:** Sugar distribution for ten *Agave* species after two-step *Agave* sugar extraction process

*Agave* species	Hydrolysate	Wash 1	Wash 2	Pretreated wash	Solids	Total carbohydrates^a^	Fructose (%)	Carbohydrates in solids (%)
*A. americana* BB	112.5	22.3	11.5	2.1	129.8	278.2	24.7	47.4
*A. americana marginata*	130.7	40.1	19.6	3.0	131.3	324.8	27.0	41.4
*A. americana gainesville*	135.3	24.5	10.7	2.6	154.7	327.8	24.7	48.0
*A. salmiana*	117.0	47.5	25.1	5.8	114.7	310.1	32.6	38.9
*A. tequilana*	70.6	29.3	13.1	6.3	208.6	327.8	18.1	65.6
*A. parryi*	87.7	33.5	13.5	7.0	198.1	339.9	19.0	60.4
*A. univitatta*	54.0	30.2	7.8	4.8	187.9	284.6	15.8	67.7
*A. lechuguilla*	36.4	8.7	3.8	4.0	250.0	302.9	5.3	83.9
*A. neomexicana*	130.9	26.6	16.5	11.8	199.6	385.3	29.8	54.9
*A. havardiana*	66.9	18.3	11.6	8.0	225.7	330.5	14.5	70.7

Sugars are glucose, xylose, galactose, arabinose, mannose, and fructose. Data is mg sugar/g dry biomass or percent content for two selected fractions

^a^Data in mg/g biomass

**Fig. 5:**
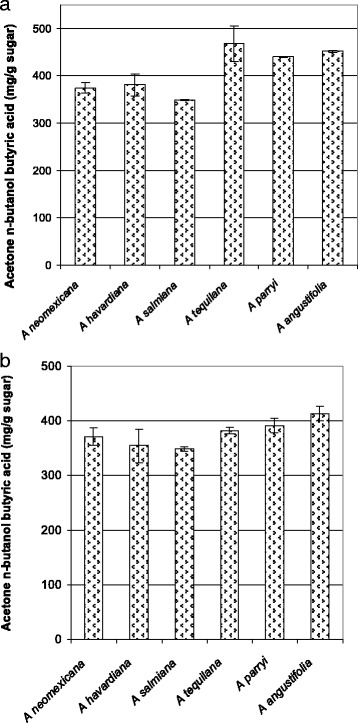
Acetone, *n*-butanol, and butyric acid (ABB) production by *C. beijerinckii* fermentation of soluble (**a**) or solids (**b**) fraction from the two-step process. **a** ABB production per gram input sugars for the neutralized 1 % acid extracted sugars (first fermentation in Fig. 3b). **b** ABB production per gram input sugars for the neutralized enzymatically hydrolyzed solids (second fermentation in Fig. 3d). Data for both are mg ABB/g sugar for duplicate 35 °C 120-hour fermentation with standard deviation

The two-step process was used to produce dual hydrolysates from five *Agave* species: *A. neomexicana, A. havardiana*, *A. salmiana*, *A. tequilana, A. parryi,* and *A. angustifolia*, followed by fermentations with *C. beijerinckii* BA101*.* As shown previously, inulinase was unneeded and omitted from the enzymatic hydrolysis of the solid in step 2 of this process. Figure 5a shows the results of the fermentation on the basis of available sugars from the neutralized 1 % sulfuric acid hydrolysis. All species produced between 349 ± 1.6 and 452 ± 2.6 mg acetone, *n*-butanol plus butyric acid (ABB)/g sugar after fermentation, with the butyric acid contribution being high from 102–220 mg/g sugar. Figure 5b shows the results of fermentation of the enzymatic hydrolysis of the solids from step 2 with yields ranging from 349 ± 4.3 to 413 ± 13.4 mg ABB/g sugar after fermentation. During the fermentation of the enzymatic hydrolysate by *C. beijerinckii* BA101, a significant portion of the carbon was left as butyric acid, higher than for earlier tests, ranging from 181 to 301 mg butyric acid/g sugar, with *A. neomexicana* showing 80 % of the fermentation product as butyric acid (322 ± 16.0 mg ABB/g sugar vs 75 ± 8.2 AB/g sugar). The raw data for Fig. 5a, b is available in Additional file 2: Table S1. Using the data from Table 2 and Fig. 5a, b, the yield of ABB/g biomass can be calculated. *A. neomexicana* has the highest level of total carbohydrates (385 mg/g biomass) and produced 144 ± 3.5 mg ABB/g biomass, higher than obtained for ethanol fermentation without pretreatment (Fig. 2a). However, ethanol conversion of the sugars from the two-step process was not available as the 1 % acid hydrolysate was not fermentable without addition of inulinase (Fig. 3b, c), and the required inulinase was not added to the solid hydrolysis during the *C. beijerinckii* testing.

The conditions for the screening process used bottles and serum vials in small scale. While beneficial for initial analysis of potential of multiple substrates with replicates, it does not permit a balanced fermentation likely provided by fully instrumented bioreactors. Also, the monitoring of the fermentation by venting the vials may have contributed to the butyric acid accumulation due to the removal of hydrogen pressure that promotes *n*-butanol production [26]. In addition, the impact of the *Agave* metabolic constituents to early termination of the conversion of butyric acid to *n*-butanol is not known. In that regard, Fig. 5a, b shows very high overall yield on sugar substrate in some cases exceeding theoretical expectation of ~410 mg AB/g glucose based upon analysis of typical simple sugars produced by “conventional” biomass. We have determined that enzymes and buffers do not support production of ABB by *C. beijerinckii* (data not shown). However, the P2 medium used is not a fully minimal medium containing 1.0 g/L yeast extract (see “Methods” section). In addition, *Agave* produce complex fructans and organic acids [4, 7] as part of their CAM metabolism, and characterization of their availability and fermentability will require extensive analysis on species by species basis. Their presence in the fermentation broth could contribute to ABB production by a metabolically versatile microorganism like *C. beijerinckii*, potentially explaining the high yield on substrate basis.

### Potential inhibitors of *Agave* sugar fermentation

It has been noted that some *Agave* carbohydrate extracts are known to be unfermentable [33]. The impact of inclusion of inulinase in *Agave* hydrolysis used for yeast fermentation is surprising yet beneficial as the unfermentable extracts from about a dozen *Agave* species can now be used for production of biofuels and other chemicals using both the abundant readily solubilized and structural carbohydrates. We have shown that the impact of the industrial inulinase preparation from Novozymes requires at least 2 days of incubation with the milled *Agave* to permit yeast fermentation even in the presence of ample fermentable sugars (Fig. 1c). Additionally, acid hydrolysis of the inulin-like molecules in *Agave* which liberates fermentable sugars does not mitigate this inhibition. This suggests it is not the complex fructans that are inhibitory. Also, the production of high levels of fermentable carbohydrates by the carbohydrase mixtures within 1 day, yet detoxification takes 2 days, suggests the removal of the inhibitor may be the result of a minor or low concentration enzymatic activity. Others recently described [31] the inability of *S. cerevisiae* to ferment the leaf juice of *A. fourcroydes* even with addition of an unspecified Sigma-Aldrich inulinase. However, *Kluyveromyces marxianus* successfully fermented the juice [31], possibly due to its inulinase production along with other enzymes [34]. Indeed, *Agave* is known to produce anti-fungal glycosylated triterpenoids called saponins that have strong anti-fungal and anti-yeast activity [33, 35–37]. Their action on fungi has been shown to be membrane permeabilization [38]. During this work, it was noticed that during yeast fermentation of *Agave* hydrolysates in the absence of solids, the lack of inulinase resulted in a clearing of the visible yeast turbidity, and no viable yeast based upon direct Petri plate tests, indicating possible cell lysis consistent with membrane disruption. Saponins can be inactivated enzymatically as shown in the pioneering work by Cira et al. [33] where a common tomato saponin, α-tomatidine, can be inactivated by a *Fusarium* tomatinase after cloning this gene into *S. cerevisiae*. When this genetically modified yeast was tested on the must (extract) of *A. tequilana* and *A. salmiana*, the fermentations produced ethanol while the wild-type yeast did not. Interestingly, these results differ from this work since in our hands the carbohydrates in *A. tequilana* never failed to fully support fermentation using two different species of *Saccharomyces* plus *C. beijerinckii*. In that regard, we have shown that *S. cerevisiae* D5A was inhibited by 50 mM α-tomatidine and resulted in clearing of turbidity similar to what was seen with *Agave* hydrolysate fermentations lacking inulinase. These results suggest the both *K. marxianus* and *A. niger,* the source of the Novozymes inulinase, produce extracellular enzyme preparations that may contain a saponinase-like activity similar to that found in *Fusarium* and other fungi, but detailed tests are required to verify this possibility. However, development and use of detoxifying enzyme preparations could benefit the expansion of yeast-based ethanol production from *Agave* species, including those growing in colder climates such as *A. neomexicana*, *A. havardiana*, and *A. parryi.*

### Potential yield of biofuels from *Agave*

Table 2 shows the sugar levels produced from the two-step conversion process ranged from 278 to 385 kg/MT biomass generated from both solubilized and structural carbohydrates. It is assumed the solubilized sugars in step 1 (Fig. 3d) are more easily converted to biofuels than the solids that require pretreatment and enzymatic hydrolysis and have the potential to be both incomplete and produce fermentation inhibitors. Using these assumptions, estimates on potential ethanol yield were calculated for *A. tequilana* plus the two US-grown *Agave* with the highest sugar levels: *A. neomexicana* and *A. parryi*. To estimate the potential ethanol yield, glucose-to-ethanol conversion of 0.51 g/g was used. The conversion efficiency for the soluble fractions (1 % hydrolysate plus washes) was (optimistically) estimated at 90 %, and for the solid fraction requiring pretreatment and enzyme hydrolysis, conversion efficiency of 75 % was used. Using these assumptions, our *A. tequilana* has the potential to produce ~170 L/MT dry biomass using data from Table 2 (70.6 + 29.3 + 13.1 kg/MT × 0.51 × 0.9 × 1.267 (L/kg)) + (6.3 + 208.6 kg/MT × 0.51 × 0.75 × 1.267) so at 25 MT/ha [5, 16], this could yield over 4200 L ethanol/ha. This yield does not match the expected yield from mature field-grown *A. tequilana* with a prominent central cone but rather this calculation is intended to provide a comparison with other *Agave* tested. For example, *A. neomexicana* from a temperate region might yield 204 L/MT dry biomass, and *A. parryi* might produce 178 L/MT dry biomass. Total plant yield (MT/ha) for these two species are speculative as the locations for growing US-based *Agave* are unknown but estimates for cultivation of *Agave* in the US have been published [19]. Also, these yields are from young plants (Table 1) and carbohydrate content may vary considerably by size of the *Agave*. However, the *A. neomexicana* plant was about 156 g wet weight (ww) and *A. tequilana* plant was 1708 g (ww), but they had similar carbohydrate levels at 385 and 328 mg/g dry biomass, respectively. Also, we found that our largest plants, *A. americana* and *A. salmiana,* had about 53–61 % *w*/*w* of their carbohydrates as solubilizable sugars (Table 2), while results with larger *Agave* leaves from Mexico had similar solubilizable sugar levels at 54–57 % *w*/*w* (Table 6 in [11]). Regardless, these results provide support for the agronomic development of multiple *Agave* species at locations optimum for each cultivar, and their biofuel production will supplement biofuels produced from bagasse of highly regulated and restricted beverage species [1]. Therefore, there is good potential to develop numerous species of *Agave* as a dedicated biofuels feedstock and provide additional sources of *Agave* biofuels from diverse semi-arid location around the globe.

## Conclusion

We have shown that the carbohydrates in multiple whole *Agave* species can support fermentation by yeast to ethanol, but in most cases, only if an industrial enzyme inulinase preparation from Novozymes is included during the hydrolysis of the *Agave* biomass. This discovery opens up the potential to use *Agave* species that are not valuable for beverage production. When included, this enzyme preparation eliminates a toxic condition that does not permit the yeast to ferment available simple sugars. The bacterium *C. beijerinckii* was not impacted by the lack of the inulinase preparation and it readily fermented the hydrolysate carbohydrates from nine *Agave* species to *n*-butanol, acetone and varying amounts of butyric acid. A two-step bioconversion procedure was developed to capture the easily solubilized simple carbohydrates, especially fructose, present in *Agave* species, while permitting later pretreatment of the structural carbohydrates needed to improved their hydrolysis and fermentation. With further testing, this dual process should maximize the amount of simple carbohydrates available for production of biofuels and other fermentation products from whole *Agave* plants. Follow-on research can include determination of the detoxification agent in the Novozymes inulinase preparation, evaluation of more mature field-grown plants (≥5 years), screening of additional *Agave* species, and complete optimization of the processing and fermentation conditions needed to fully convert all available fermentable carbohydrates in non-beverage *Agave*, such as cold-tolerant *A. neomexicana,* to biofuels and biochemicals such as ethanol and *n*-butanol.

## Methods

*Agave* plants were obtained from commercial nurseries with sizes between large plants (2.5 kg) to small (under 0.1 kg) (Table 1) and maintained in containers. *A.americana* Big Blue, *A americana* var. *marginata*, *A. americana* var. *gainesville*, *A. salmiana* var. *ferox*, *A.tequilana. A. decipiens, A. ghiesbreghtii*, *A. univitatta* var. *truncata*, *A. parryi* var. *compacta*, and *A. angustifolia* were obtained from Notestein’s Nursery, (Southerngardening.org), Gainesville, FL, USA. *A. neomexicana, A. havardiana*, and *A. lechuguilla* were obtained from Desert Sage Nursery, Kingman, AZ, USA. The *Agave* plants were grown in ambient light and harvested in the afternoon (after 2 p.m.) to permit completion of the CAM cycle. The plants were chopped into >1 cm pieces and immediately held at 45 °C until dry while avoiding excessive drying. Dried biomass was milled through a 20-mesh Wiley mill and stored sealed at room temperature until used. Biomass weights used in experimentation are on a dry basis (db), excluding plant weights (Table 1).

*S. cerevisiae* D5A was obtained from the ATCC (Manassas, VA, USA) as ATCC 200062. *S. bayanus* Lalvin EC-1118 from Lallemand Inc. was obtained at Ferment Station Inc., Knoxville, TN, USA. *C. beijerinckii* BA101 was obtained from the ATCC as No. PTA-1550.

Simultaneous saccharification and fermentation (SSF), separate hydrolysis and fermentation (SHF), and pretreatment were conducted with *Agave* biomass as described previously [23, 39]. Essentially, SSF fermentations contained selected weights of dry biomass mixed with water, buffer, enzymes, nutrients, and a bacterial antibiotic. A typical SSF protocol is 2 g biomass mixed with water needed to reach 40 mL final, plus 2 mL 1 M sodium citrate buffer pH 4.8, followed by autoclaving for 30 min. After cooling, selected levels of enzymes (see below), 2 mL 10 % yeast extract, 1.0 mL overnight culture of selected fermentation microorganism and streptomycin to 62.5 μg/mL (to minimize contamination) are added, and initial weight is recorded followed by incubation at selected temperature with shaking, depending upon the microorganism. Containers used were 70 mL Septi-Chek glass bottles with screw caps and rubber seals (Becton Dickinson, Franklin Lakes, NJ, USA; http://www.bd.com). These have been discontinued so serum vials with stoppers can be used to maintain anaerobic conditions. SHF differs with the preparation lacking the microorganism, yeast extract, and antibiotic until after a hydrolysis process for multiple days at selected temperatures. *Agave* hydrolysis typically was conducted at 50 °C for 4 days shaking at 100 rpm. The SSF fermentation is initiated after cooling by addition of the fermentation microorganism, yeast extract, and antibiotic, as above, followed by incubation at selected temperature with shaking, depending upon the microorganism. At the end of the fermentation, the solid-containing broth was harvested and sugar levels were analyzed after solids were separated from the free liquid as described elsewhere [23] and below. More method details are available at [40].

Mild hydrolysis was conducted with unpretreated *Agave* which was mixed with 1 % sulfuric acid in high pressure tubes (Chemglass, Vineland, NJ, USA) and heated in a fluidized sand bath (Omega FSB1: Techne Co., Princeton, NJ, USA) at 100 °C for 30 min followed by cooling to room temperature. Biomass was washed with water and centrifuged in BD Falcon 50 mL tubes at 8000 rpm for 20 min in a Sorvall Legend RT centrifuge (Thermo Scientific, Waltham, MA, USA). Total carbohydrate composition was determined by this mild hydrolysis of readily solubilized carbohydrates described above followed by HPLC analysis [23, 39]. Composition of the solids was determined by enzymatic hydrolysis after pretreatment using the SHF hydrolysis protocol [39]. Total fermentable sugars refers to the major simple sugars that *S. cerevisiae* D5A can utilize: glucose, fructose, galactose, and mannose, while *C. beijerinckii* can also utilize cellobiose, arabinose, and xylose.

*n*-Butanol fermentations used double-strength sterile anaerobic P2 medium [41] with 2.0 g/L yeast extract into which hydrolysate, water, and typically 2.5 % *v*/*v* of an overnight inoculum of *C. beijerinckii* BA101 was added to an overall volume equal to the 2× P2 volume yielding 1× P2 medium. Streptomycin can be used as above since *C. beijerinckii* BA101 is resistant to this antibiotic. All fermentations were set up in an anaerobic chamber (Coy Laboratory Products, Grass Lake, MI, USA) with 125 mL serum bottles sealed with black butyl rubber stoppers (Geo-Microbial Technologies, Inc., Ochelata, OK, USA) with a crimp seal accompanied by proper degassing to attain anaerobic conditions [39, 42]. Fermentations were conducted with 80 rpm shaking at 35 °C. Fermentation progress was monitored by periodic venting in an anaerobic chamber with a 1.5′′ 27 gauge needles and subsequent weight loss determination. Fermentation with strict anaerobic bacteria requires proper handling to maintain anaerobic conditions [39, 42].

Polyfructose hydrolysis tests: quadruplicate pairs of 2-g samples of biomass were suspended in water at 10 % *w*/*w*, autoclaved, and H_2_SO_4_ was added to obtain final concentration of 1 % *w*/*w*. Two pairs were heated to 100 °C. for 30 min in a sand bath using glass pressure tubes (Chemglass, Vineland, NJ, USA) to fully hydrolyze polyfuctose molecules as per Penner [30]. The others were kept at room temperature. All samples were neutralized to approximately pH 5 judged by pH paper using calcium carbonate. One heated pair was immediately prepared for SSF processing without inulinase addition while the three remaining samples were prepared for hydrolysis of the solids, both as described below. One pair included inulinase during hydrolysis phase of the SHF process at the levels described below.

Multifect Pectinase was provided gratis by Genencor International, a division of DuPont. Novozymes cellulase Ctec2 and hemicellulase Htec were supplied gratis by Novozymes. Ctec2 contained 295.1 mg protein/mL as determined by protein assay (K. Yee, personal communication). Novozymes inulinase 690 #I6285 and ß-glucosidase 188 #C6105 were purchased from Sigma-Aldrich Chemical Co, St Louis, MO, USA. Cellulase was added for both SSF and SHF at high levels to provide ample enzyme for these different substrates using 0.1 mL of Ctec2 per gram of dry biomass, and accessory enzymes (pectinase, Htec2, ß-glucosidase 188) were added at 25 % level (*v*/*v*) of the cellulase. Inulinase was added at 32.9 INU/g biomass, unless stated otherwise, and the Novozymes inulinase 690 has 329 INU/mL. One Inulinase Unit (INU) is the amount of enzyme which produces 1 μmol reducing carbohydrate (calculated as glucose) per minute at 40 °C.

## Additional files

Additional file 1: Figure S1.
**ABB fermentation time course for three** ***Agave*** **species from Fig.** 2**b**. Examples of SHF progress for three *Agave* species with either active or inactive inulinase in the hydrolysis. Shown is weight loss with standard deviation. 35 °C 162 hours fermentation, *n* = 2. Additional file 2: Table S1.
**Raw data for the** ***C. beijerinckii*** **fermentation shown in Figs.** 2 **b and** 5**a, b**. Fermentations were conducted in duplicate and used to calculate the standard deviation. ABB = acetone, *n*-butanol, butyric acid. Active is native inulinase; inactivated is heated inulinase. Additional file 3: Table S2.
**Percentage distribution of simple sugars in ten** ***Agave*** **after processing with the two-step process.** The data is shown graphically in Fig. 4.

## Abbreviations

CAM: crassulacean acid metabolism
SSF: simultaneous saccharification and fermentation
SHF: separate hydrolysis and fermentation
INU: inulinase unit
prtd: pretreated
res: residual
sug: sugar
glu: glucose
xyl: xylose
gal: galactose
fru: fructose
mann: mannose
arab: arabinose
carb: carbohydrate
ABB: acetone, *n*-butanol, butyric acid
bm: biomass
db: dry basis
ww: wet weight
inul: inulinase enzyme
heat: heated inulinase enzyme
A Amer BB: *A. americana* Big Blue
A amer marg: *A. americana* marginata
A amer gain: *A. americana* gainesville
