# Supplementary material for: Development of Agave as a dedicated biomass source: production of biofuels from whole plants
Source: Biotechnol Biofuels. 2015 May 30;8:79. doi: 10.1186/s13068-015-0261-8 (PMC4459669; doi:10.1186/s13068-015-0261-8)
Supplement: Additional file 1: Figure S1. — ABB fermentation time course for three Agave species from Fig. 2b. Examples of SHF progress for three Agave species with either active or inactive inulinase in the hydrolysis. Shown is weight loss with standard deviation. 35 °C 162 hours fermentation, n = 2. [file 13068_2015_261_MOESM1_ESM.doc]

**Figure S1 ABB fermentation time course for three *Agave* species from Figure 2b.**
